# The roles of protocadherin-7 in colorectal cancer cells on cell proliferation and its chemoresistance

**DOI:** 10.3389/fphar.2023.1072033

**Published:** 2023-03-30

**Authors:** Zhibao Zheng, Na Luan, Kai Tu, Feiyan Liu, Jianwei Wang, Jianguo Sun

**Affiliations:** ^1^ Department of Surgical Oncology, Taizhou Central Hospital (Taizhou University Hospital), Taizhou, Zhejiang, China; ^2^ Department of Colorectal Surgery and Oncology, The Second Affiliated Hospital, Zhejiang University School of Medicine, Hangzhou, China; ^3^ College of Life Sciences, Zhejiang University, Hangzhou, China

**Keywords:** protocadherin-7, Mcl-1, chemoresistance, Wnt/beta-catenin, colorectal cancer

## Abstract

Despite the high mutation frequencies of *KRAS*, *NRAS*, and *BRAF* in colorectal cancer (CRC), there are no effective and reliable inhibitors for these biomarkers. Protocadherin-7 (PCDH7) is regarded as a potentially targetable surface molecule in cancer cells and plays an important role in their proliferation, metastasis, and drug resistance. However, the roles and underlying mechanisms of PCDH7 in CRC remain unclear. In the current study, we found that different colorectal cancer cells expressed PCDH7 over a wide range. The levels of PCDH7 expression were positively associated with cell proliferation and drug resistance in CRC cells but negatively correlated with the potential for cell migration and invasion. Our data indicated that PCDH7 mediated the resistance of CRC cells to ABT-263 (a small-molecule Bcl-2 inhibitor that induces apoptosis) by inhibiting cell apoptosis, which was supported by the downregulation of caspase-3, caspase-9, and PARP cleavage. We found that PCDH7 effectively promoted Mcl-1 expression at both mRNA and protein levels. Furthermore, PCDH7 activated the Wnt signaling pathway, which was confirmed by the increase in β-catenin and c-Myc expression. Finally, and notably, S63845, a novel Mcl-1 inhibitor, not only effectively attenuated the inhibitory effect of PCDH7 on cell apoptosis induced by ABT-263 *in vitro* but also sensitized PCDH7-overexpressed CRC cell-derived xenografts to ABT-263 *in vivo*. Taken together, although PCDH7 inhibited the migration and invasion of CRC cells, it could facilitate the development of drug resistance in colorectal cancer cells by positively modulating Mcl-1 expression. The application of the Mcl-1 inhibitor S63845 could be a potential strategy for CRC chemotherapy, especially in CRC with high levels of PCDH7.

## 1 Introduction

Colorectal cancer (CRC) is one of the most lethal malignancies worldwide (Bray et al., 2018). In the United States, CRC has been the second-most common cause of cancer-related death for years because of the aging population and dietary habits (Siegel et al., 2020).

Although apparent declining trends in the incidence and mortality in the older population in high-income countries seem to be from a nationwide screening program and the wide application of colonoscopy in general (Connell et al., 2017), the rapid increase in both CRC incidence and mortality is ongoing in several developing countries, particularly in Eastern Europe, Asia, and South America (Dekker et al., 2019). Thus far, surgery is still the cornerstone of colorectal cancer treatment, and the combination of chemotherapy and molecular targeted therapy gradually contributes significantly to the improvement of patients’ overall survival and quality of life (Kopetz, 2019). Numerous studies have demonstrated that overexpression of EGFR and alteration of signaling pathways could trigger and/or accelerate the progression of CRC, which assists in the treatment against EGFR using monoclonal antibodies (e.g., cetuximab and panitumumab) in CRC-targeted therapies. Although high mutation frequencies of *KRAS*, *NRAS*, and *BRAF* are present in CRC, there are no effective and reliable inhibitors of these biomarkers. Monotherapy with a *BRAF* inhibitor only yields a response in approximately 5% of CRC patients with the *BRAF* ^*V600E*^ mutation, which is one of the prominent mutations in approximately 10% of CRC patients (Venderbosch et al., 2014). RAS (including *KRAS* and *NRAS*) inhibitors have also been investigated for decades and, unfortunately, are still so elusive that RAS is charted as an undruggable target (Moore et al., 2020). Therefore, discovery of the underlying molecular mechanisms facilitating the development and metastatic progression of CRC would benefit the exploration of novel therapeutic targets.

Protocadherin-7 (PCDH7) belongs to the protocadherin family and was first found to be prominently expressed in brain tissue (Yoshida et al., 1998). Recently, it has been demonstrated that PCDH7 can regulate osteoclastogenesis by promoting cell–cell fusion and maintenance of bone homeostasis (Kim et al., 2020). Furthermore, PCDH7 has multiple biological functions in various cancers. PCDH7 was identified as one of the top-ranking genes associated with breast cancer metastasis to the brain. Reduction of PCDH7 in the metastatic breast cancer cell line MDA-MB-231 effectively inhibits cell proliferation, migration, and invasion *in vitro* (Li et al., 2013). In lung cancer, the enforced PCDH7 expression significantly accelerates lung tumorigenesis in mice harboring the *Kras* ^*G12D*^ mutation and potentiates MAPK pathway activation (Zhou et al., 2017; Zhou et al., 2019). In contrast to the promotive effects of PCDH7 in breast and lung cancer, downregulated PCDH7 correlates with advanced grade, larger tumor size, and poorer overall survival in bladder cancer and gastric cancer patients (Lin et al., 2016; Chen et al., 2017).

In the present study, we found that PCDH7 conferred higher cell proliferation potential and stronger chemoresistance in colorectal cancer cells both *in vitro* and *in vivo* by promoting MCL-1 expression. Our data provide novel insights into the underlying mechanism of the functions of PCDH7 in CRC progression, which might also help us in developing a new therapeutic strategy for CRC by targeting this molecule.

## 2 Methods and materials

### 2.1 Bioinformatics analysis of PCDH7 in colorectal cancer

Sequencing data of colorectal cancer in the TCGA database were downloaded. The correlation between PCDH7 expression and its clinical parameters in CRC patients was analyzed using two-sample *t*-test.

### 2.2 Cell lines and cell cultures

The colorectal cancer cell lines in this study, including LS411N (colorectal carcinoma), DLD1 (colon adenocarcinoma), RKO (colon carcinoma), HCT116 (colon adenocarcinoma), SW620 (Caucasian colon adenocarcinoma), HT29 (Caucasian colon adenocarcinoma), and SW480 (colon adenocarcinoma), were purchased from American Type Culture Collection (ATCC), and all cell lines are *KRAS* mutants. All cells were cultured in Dulbecco’s modified Eagle’s medium (DMEM) (HyClone) supplemented with 10% fetal bovine serum (FBS) (HyClone), 1% penicillin (100 units/mL), and streptomycin (100 μg/mL) (Sigma). These cells were placed in a cell culture incubator supplied with 5% CO_2_, maintained at 37°C, and passaged at ≥80% confluence by trypsinization (Gibco). All experiments were performed when the cells were in the logarithmic growth phase.

### 2.3 Western blotting and the antibodies

Cells were lysed with RIPA buffer containing 50 mM Tris-HCl (pH 8.0), 150 mM NaCl, 2 mM EDTA (pH 8.0), 0.1% Triton-X 100, 0.5% sodium deoxycholate, 0.1% sodium dodecyl sulfate, and 1% protease inhibitor (Sigma). The lysate was incubated on ice for 10 min, the supernatant was collected after centrifugation at high speed, and the cell lysate was prepared with 2× SDS loading buffer after denaturation. Gel electrophoresis was performed on an 8%–15% gradient acrylamide gel to separate the proteins, which were then transferred onto a PVDF membrane (Millipore). After blocking with 5% fat-free milk in PBST for 2 h at room temperature, the PVDF membranes were incubated with the corresponding primary antibodies at 4°C overnight. The predicted proteins were detected using the corresponding secondary antibody-conjugated HRP. Protein bands were detected using an ECL chemiluminescence reaction kit (Thermo Fisher Scientific). ImageJ software was used for quantitative analysis.

Antibodies for immunoblotting included anti-PCDH7 (Santa Cruz, sc-517042, 1:500), anti-PARP (CST, 9532S, 1:1,000), anti-caspase 9 (CST, 9502, 1:1,000), anti-caspase 3 (CST, 9668, 1:1,000), anti-Mcl-1 (CST, 5453, 1:1,000), anti-β-catenin (CST, 8480, 1:1,000), anti-p-GSK-3β (CST, 9421, 1:1,000), anti-c-Myc (CST, 5605, 1:1,000), and mouse anti-β-actin (Sigma, A5441) diluted at 1:5,000.

### 2.4 Generation of PCDH7 knockdown cell lines and PCDH7 overexpression cell lines

PCDH7-siRNAs were purchased from GemmaPharma Bio. (Shanghai, China), and the human PCDH7 overexpression plasmid was purchased from Molecular Detection Bio (Hangzhou, China). The plasmid sequences were verified by sequencing. Transfection of siRNA or the PCDH7 overexpression plasmid was performed using Lipofectamine 3000 (Invitrogen) according to the manufacturer’s protocol. Prior to transfection, the cells were seeded into 6-well plates at appropriate densities and cultured for 24 h in a complete medium. For PCDH7 cells stably overexpressing LS411N for animal experiments, G418 was used for cell selection. Cells were collected in the experiments designed to perform the corresponding experiments. The efficiency of overexpression or knockdown was evaluated by Western blotting of transfected cells after 48 h of transfection.

### 2.5 MTT assay

Cell proliferation was evaluated using the Cell Proliferation Kit I (MTT) (Roche) according to the manufacturer’s protocol. Briefly, different colorectal cancer cells overexpressing PCDH7 or PCDH7 knockdown were collected 24 h after transfection, seeded into 96-well plates at an appropriate density (3 × 103/well), and cultured overnight to allow cell adherence. At the indicated time points, the corresponding cells were treated with 50 μL MTT reagent (final concentration 0.5 mg/mL) and incubated at 37°C for up to 4 h, followed by the addition of 150 μL of DMSO. Cell viability was checked by measuring the absorbance at 570 nm using a microplate reader (Molecular Devices).

For cytotoxicity detection, cancer cells were seeded into 96-well plates at a suitable density and treated with the indicated concentrations of ABT-263 or cisplatin for 24 and 48 h prior to performing the MTT assay. ABT-263 was diluted in DMSO to prepare the mother solution at 100 mg/mL and then diluted into the actual working concentration with the culture medium. Cisplatin was diluted in DMSO to prepare the mother solution at 5 mg/mL and then diluted into the actual working concentration with the culture medium.

Cell proliferation rate % = (experimental group OD value−0 day OD value)/(control group OD value−0 day OD value) * 100%; cell relative survival rate % = experimental group OD value/control group OD value * 100%

### 2.6 Cell migration and invasion assays

Chambers (8-mm pore, BD Falcon, BD Biosciences) with or without Matrigel (BD Biosciences) were used to investigate invasion and migration, respectively. Colorectal cancer cells were suspended in a serum-free medium and seeded into the chambers. These chambers were then placed in 24-well plates filled with 600 μL of the medium containing 20% FBS as an attractant. After 24 h of incubation at 37°C with 5% CO_2_, the cells on the upper side were removed, and the migrated or invaded cells on the underside of the membrane were fixed and stained with 0.1% crystal violet for 30 min at 37°C. After washing twice with PBS, cells on the lower membrane were counted in three independent areas.

### 2.7 Apoptosis detection by flow cytometry

Apoptosis detection was performed using the FITC Annexin V Apoptosis Kit (eBioscience), according to the manufacturer’s instructions. Briefly, the treated colorectal cancer cells were collected, and 1 × 10^6^ cells were resuspended in 500 μL 1× binding buffer containing 5 μL FITC-labeled Annexin V and 5 μL PI solution. The cells were gently vortexed and incubated for 15 min at room temperature in the dark, followed by flow cytometry (BD, Fortessa). The proportion of apoptosis refers to the sum of Annexin V-positive cells.

### 2.8 RNA isolation and qPCR

Total cellular RNA was extracted using TRIzol reagent (Invitrogen), according to the manufacturer’s instructions. cDNA was synthesized using total RNA as a template and Promega M-MLV reverse transcriptase. Real-time PCR was performed on an ABI 7500 Fast Real-Time PCR system (Applied Bioscience) with Power SYBR Green PCR Master Mix (Applied Biosystems), according to the manufacturer’s instructions. Primers used were as follows: Mcl-1, forward primer 5′-AAA​GCC​TGT​CTG​CCA​AAT-3′; reverse primer 5′-TTA​GAC​CAC​CTG​CCT​CCT-3′; β-actin, forward primer 5′-ACACCCCAGCCAT GTACGTT-3′; reverse primer 5′-TCACCGGA GTCCATCACGAT-3′. The specificity of these primers was determined using a melting curve. The relative expression of the genes was analyzed using the 2-△△CT method, and β-actin was used as a control.

### 2.9 Tumor growth inhibition *in vivo*

Four-week-old female athymic BALB/c mice were subcutaneously injected with 5 × 10^6^ LS411N cells. After a solid tumor formed and grew to approximately 80–100 mm^3^, the tumor-bearing mice were randomized into four groups of four mice each. Control mice were intravenously administered with saline. The treatment groups were injected intravenously with S63845 (5 mg/kg body weight) alone or ABT-263 (50 mg/kg for each mouse) orally, alone or in combination once every 2 days for 12 days. All animal experiments were approved by the Animal Experimentation Ethics Committee of Zhejiang University.

### 2.10 Statistical analyses

All data are expressed as mean ± standard deviation (mean ± SD). Data analysis was performed using GraphPad Prism 7. All experiments were repeated thrice. Differences between groups were analyzed using the paired Student’s *t*-test. *p* < 0.05 was considered to indicate statistical significance.

## 3 Results

### 3.1 The clinical significance of PCDH7 in colorectal cancer and its basic expression in colorectal cancer cells

**FIGURE 1:**
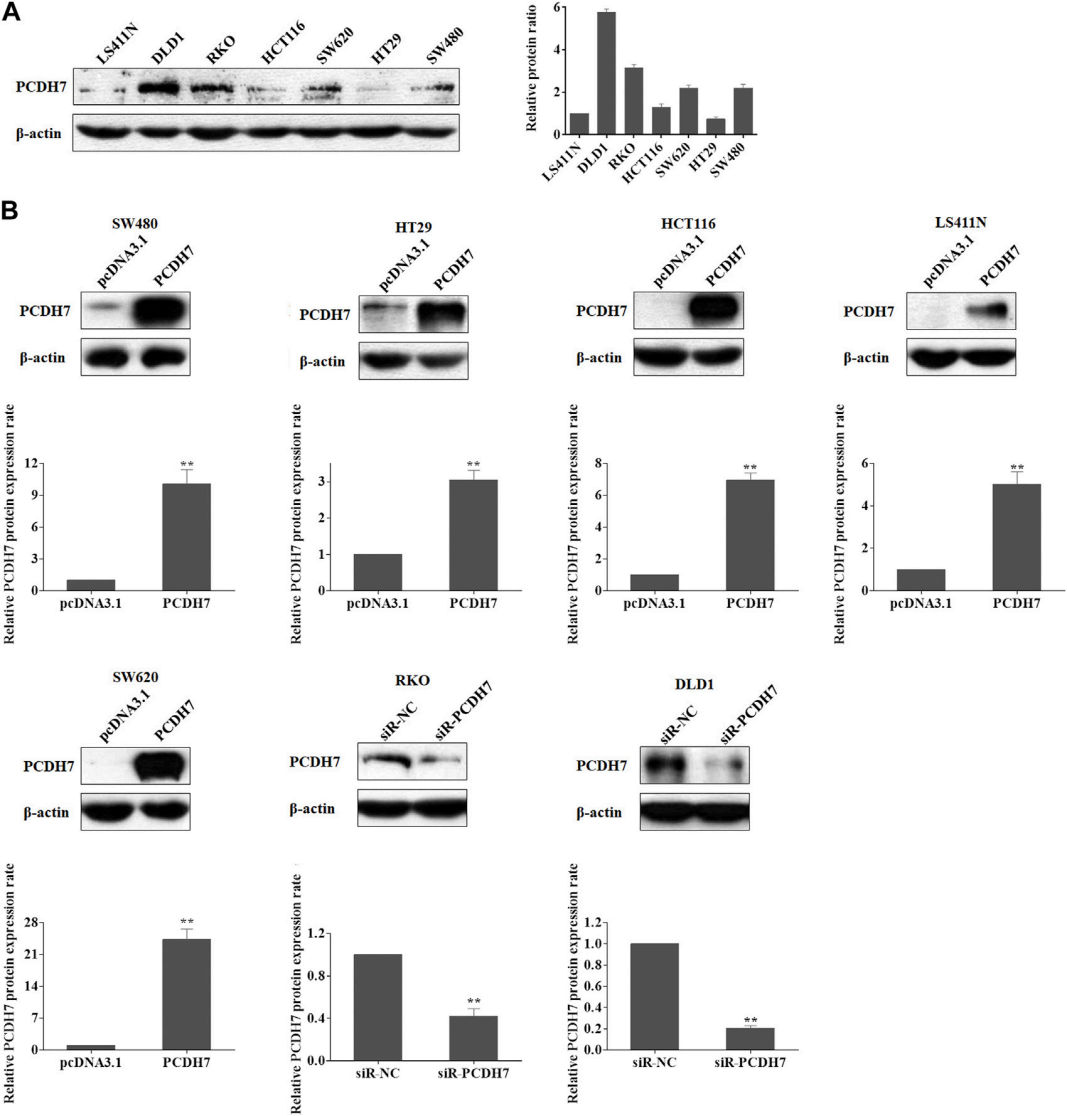
Expression of PCDH7 in colorectal cancer cell lines. **(A)** Expressions of PCDH7 protein were examined in LS411N, DLD1, RKO, HCT116, SW620, HT29, and SW480 using Western blot assay. **(B)** Overexpression of PCDH7 in SW480, HT29, HCT116, LS411N, and SW620 cells transfected with the overexpression plasmid for 24 h and the silencing of PCDH7 in DLD1 and RKO cells by siRNA transfection for 24 h were verified by Western blot assays. Data are presented as mean ± SD for three separate experiments. **p < 0.01 *vs.* control.

We first analyzed the correlation between PP4R1 expression and the clinical pathological features in 440 patients with colorectal cancer. As shown in Table 1, significant associations were obtained between PCDH7 expression and age (*p* = 0.007), T classification (*p* = 0.001), N classification (*p* = 0.003), and stage (*p* = 0.004). To investigate the potential functions of PCDH7 in colorectal cancer, we initially used Western blotting to detect the abundance of PCDH7 in different colorectal tumor cell lines, including LS411N, DLD1, RKO, HCT116, SW620, HT29, and SW480. Our data indicated that DLD1 and RKO cells expressed the highest levels of PCDH7 protein. Moderate levels of the protein were observed in SW620 and SW480 cells, whereas relatively low levels of the protein were expressed in LS411N, HCT116, and HT29 cells (Figure 1A). To characterize the potential effects of PCDH7 on colorectal cancer, we successfully induced the expression of PCDH7 in SW480, HT29, HCT116, LS411N, and SW620 cells using an overexpression plasmid, while decreasing PCDH7 expression in DLD1 and RKO cells by transfection with a siRNA against PCDH7 (Figure 1B).

**TABLE 1:** Association between PCDH7 expression and clinical pathological features in colorectal cancer patients.

Character	Level	Low expression of PCDH7	High expression of PCDH7	P
Age	≤60	84	43	0.007
	>60	244	69	
Gender	Male	64	29	0.118
	Female	264	83	
T	Tis	1	0	0.001
	T1	7	2	
	T2	65	9	
	T3	219	82	
	T4	36	19	
N	N0	205	56	0.003
	N1	72	29	
	N2	51	27	
	N3	—	—	
M	M0	286	90	0.071
	M1	42	22	
Stage	Ⅰ	65	11	0.004
	Ⅱ	133	44	
	Ⅲ	88	35	
	Ⅳ	42	22	

### 3.2 The effects of PCDH7 in colon cancer cell proliferation

**FIGURE 2:**
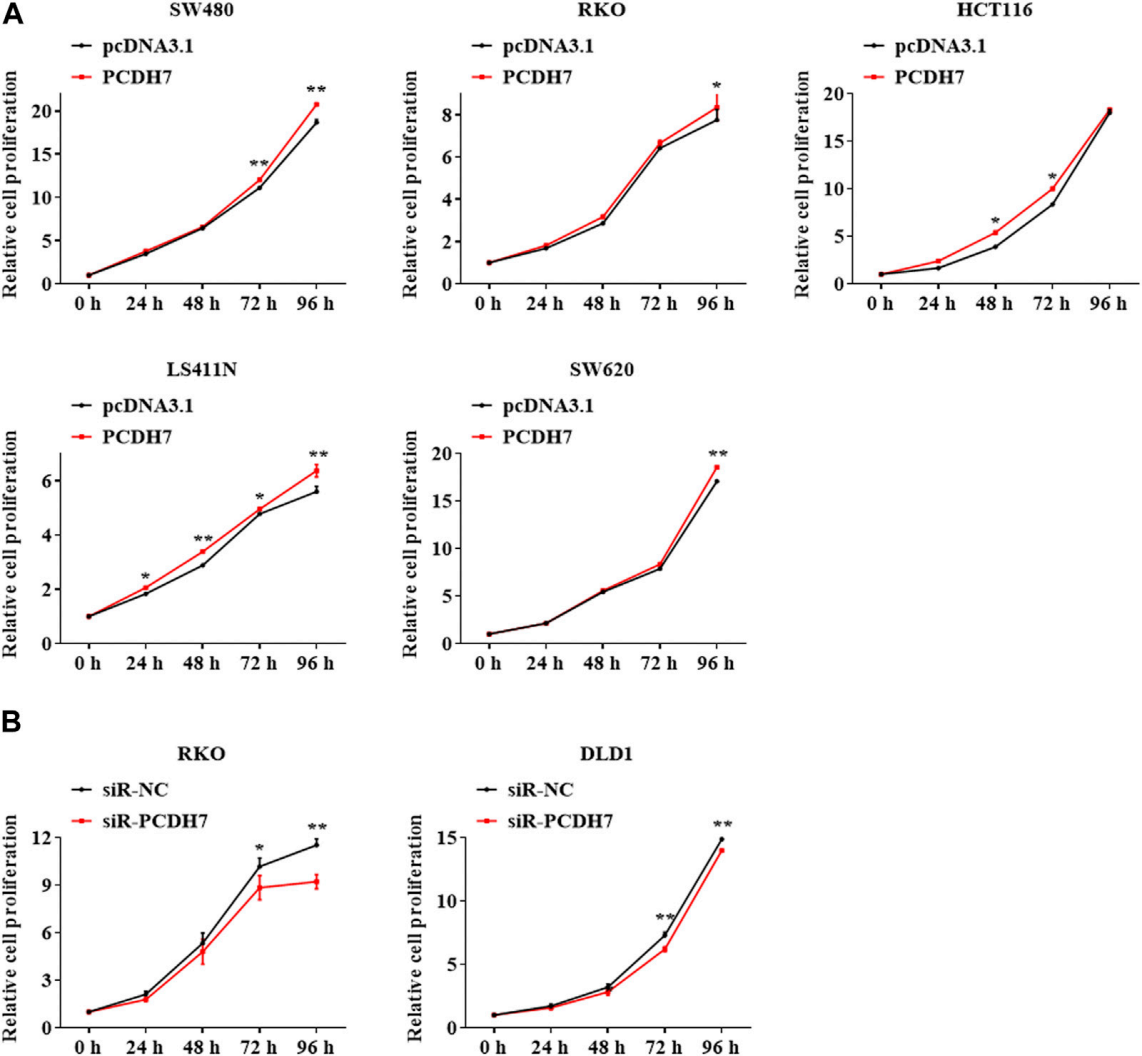
Effects of PCDH7 in colon cancer cell proliferation. **(A)** MTT assays were performed to detect the cell proliferation of SW480, HT29, HCT116, LS411N, and SW620 cells transfected with PCDH7 overexpression plasmid or empty vector pcDNA3.1 for 24 h. **(B)** Cell proliferation of RKO and DLD1 cells with PCDH7 silencing transfected with siRNA for 24 h was examined by MTT assays. Data are presented as mean ± SD for three separate experiments. **p < 0.01 *vs.* control.

Cell proliferation was detected in colorectal cancer cell lines by PCDH7 overexpression or silencing using the MTT assay. Our data revealed that the expression of PCDH7 was positively associated with the proliferation potential of CRC cells. SW480 cells overexpressing PCDH7 showed more robust proliferation than control cells transfected with the corresponding empty vector. Similar results were also observed in other cell lines, including HT29, HCT116, LS411N, and SW620 cells (Figure 2A). In contrast, PCDH7 knockdown in DLD1 and RKO cells effectively decelerated their proliferation (Figure 2B). In summary, our data implied that PCDH7 could potentially play a role in colorectal cancer progression by accelerating cancer cell proliferation.

### 3.3 The potential roles of PCDH7 in CRC cell metastasis

**FIGURE 3:**
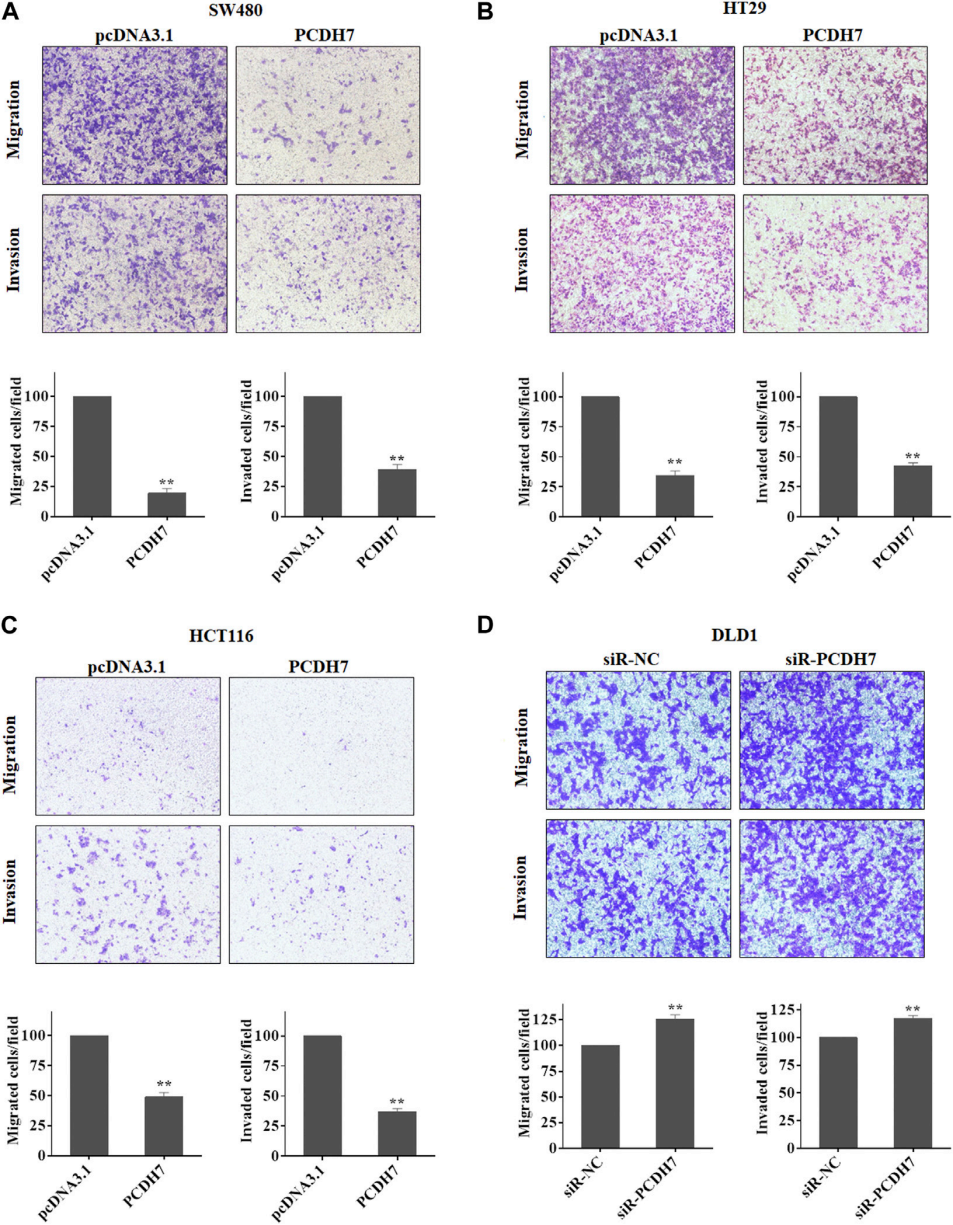
Roles of PCDH7 in CRC cell migration and invasion. **(A–C)** Transwell assays were used to determine the potentials of cell migration and invasion in SW480, HT29, and HCT116 cells with PCDH7 overexpression. **(D)** Transwell assays were performed to detect cell migration and invasion in DLD1 cells with PCDH7 knockdown. Data are presented as mean ± SD for three separate experiments. **p < 0.01 *vs.* control.

Given the widely known role of protocadherin in cell-in-cell structure to mediate cancer cell metastasis, we further determined whether PCDH7 could contribute to colorectal cancer metastasis by regulating cancer cell migration and invasion. Interestingly, our data from transwell experiments indicated that overexpression of PCDH7 in SW480, HT29, and HCT116 cells significantly weakened their migration and invasion (Figures 3A–C). To further verify the role of PCDH7 in CRC cell migration and invasion, the transwell assay was performed in CRC cells with PCDH7 silencing. As shown in Figure 3D, downregulation of PCDH7 expression significantly promoted the migration and invasion of DLD1 cells. Thus, these results indicated that PCDH7 negatively regulates colorectal cancer cell migration and invasion rather than cell proliferation.

### 3.4 PCDH7 confers drug resistance of colorectal cancer cells to ABT-263 and cisplatin

**FIGURE 4:**
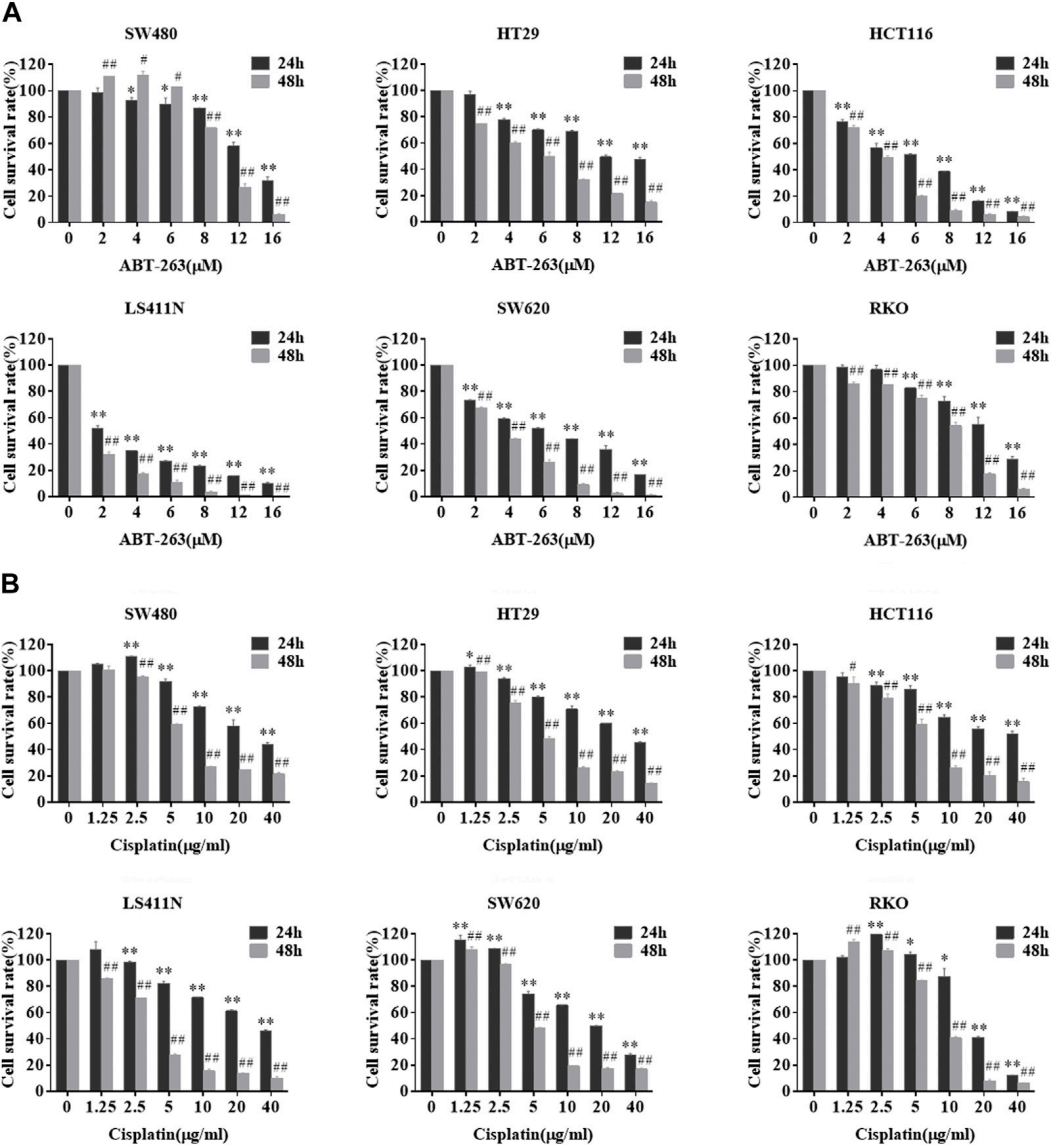
Sensitivity of different colorectal cancer cells to ABT-263 and cisplatin. After treating CRC cell lines with different concentrations of ABT-263 **(A)** or cisplatin **(B)** for 24 or 48 h, MTT assay was used to determine the cell viability to confirm the cytotoxicity of the two drugs. Data are presented as mean ± SD for three separate experiments. *, #*p* < 0.05; **, ##*p* < 0.01 *vs.* control.

Next, we investigated whether PCDH7 contributes to the development of drug resistance in CRC cells. First, we measured the sensitivity of different colorectal cancer cell lines to ABT-263 and cisplatin. ABT-263, an orally available, selective inhibitor of the anti-apoptotic BCL-2 family proteins, including Bcl-2, Bcl-XL, and Mcl-1, has been demonstrated to possess antitumor activity against various types of cancers and is in clinical trials (Lever and Fergason-Cantrell, 2019; Ohgino et al., 2020). Cisplatin, another well-known anti-cancer drug employed in treating different solid tumors, generates DNA lesions to activate the DNA damage response and induce apoptosis (Galluzzi et al., 2012). Here, we observed that both drugs effectively inhibited the viability of SW480, HT29, HCT116, LS411N, SW620, and RKO cells in a dose- and time-dependent manner (Figure 4).

**FIGURE 5:**
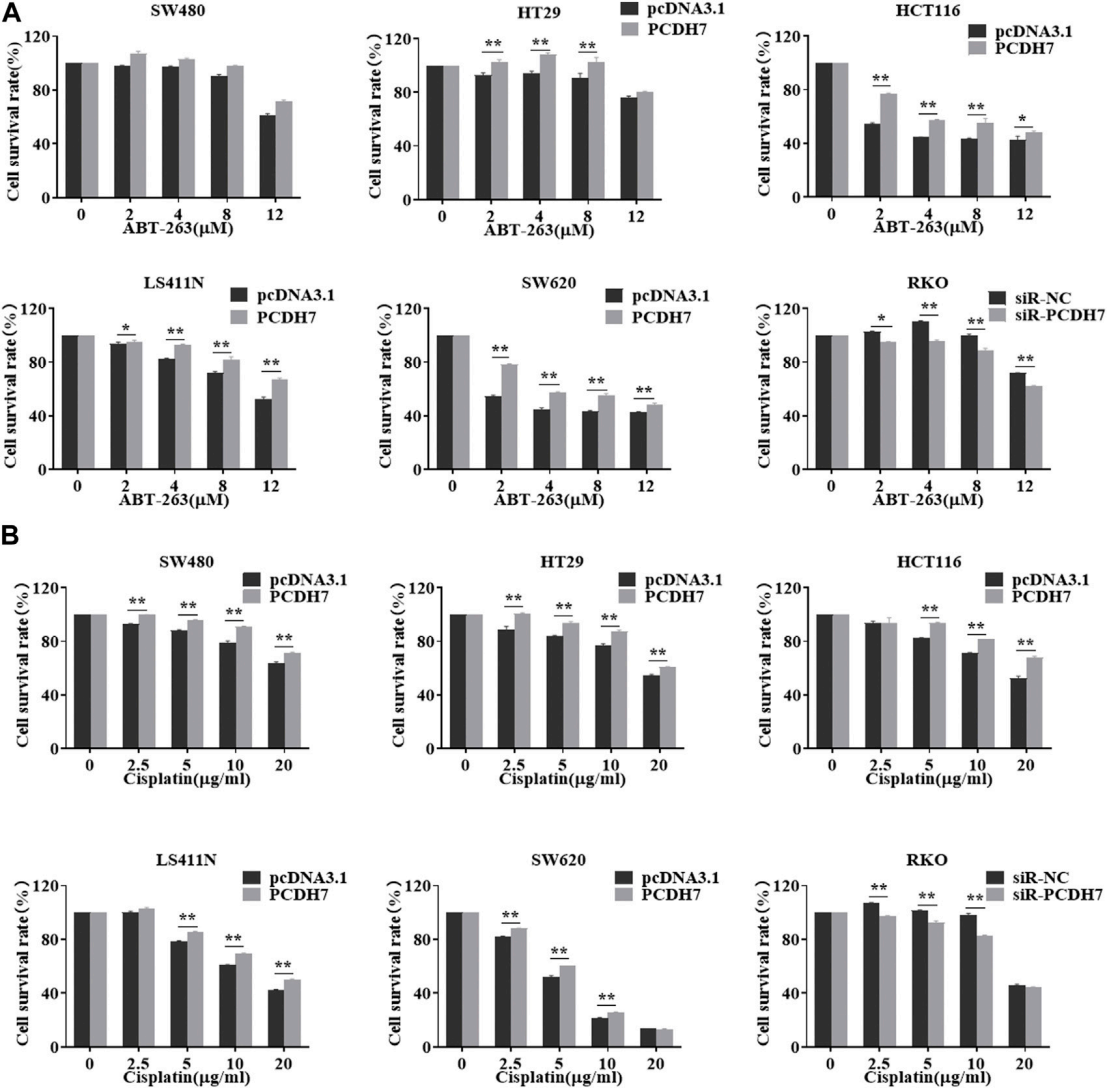
Effects of PCDH7 on the resistance of colorectal cancer cells. **(A)** MTT assays were performed to determine the cell viabilities of SW480, HT29, HCT116, LS411N, SW620, and RKO cells with the treatment of ABT-263 under PCDH7 overexpression or silencing. **(B)** MTT assays were performed to determine the cell viabilities of SW480, HT29, HCT116, LS411N, SW620, and RKO cells with cisplatin treatment under PCDH7 overexpression or silencing. Data are presented as mean ± SD for three separate experiments. *p < 0.05; **p < 0.01 *vs.* control.

To further examine the potential effect of PCDH7 on drug resistance in colorectal cancer, we performed gain- and loss-of-function experiments by overexpressing or knocking down PCDH7 in colorectal cancer cells. We found that the overexpression of PCDH7 in SW480, RKO, HCT116, LS411N, and SW620 cells significantly promoted their survival under either ABT-263 or cisplatin treatment (Figure 5). Conversely, the decrease in PCDH7 expression in RKO cells led to remarkably increased sensitivity to ABT-263 and cisplatin compared to that in control cells (Figure 5). Together, these results revealed that abundant PCDH7 enhances the resistance of colorectal cancer cells to chemotherapeutic drug treatment to facilitate cell viability, suggesting that PCDH7 is a potential regulatory target for colorectal cancer chemotherapy.

### 3.5 PCDH7 enhanced drug resistance of CRC cells to ABT-263 by inhibiting apoptosis

**FIGURE 6:**
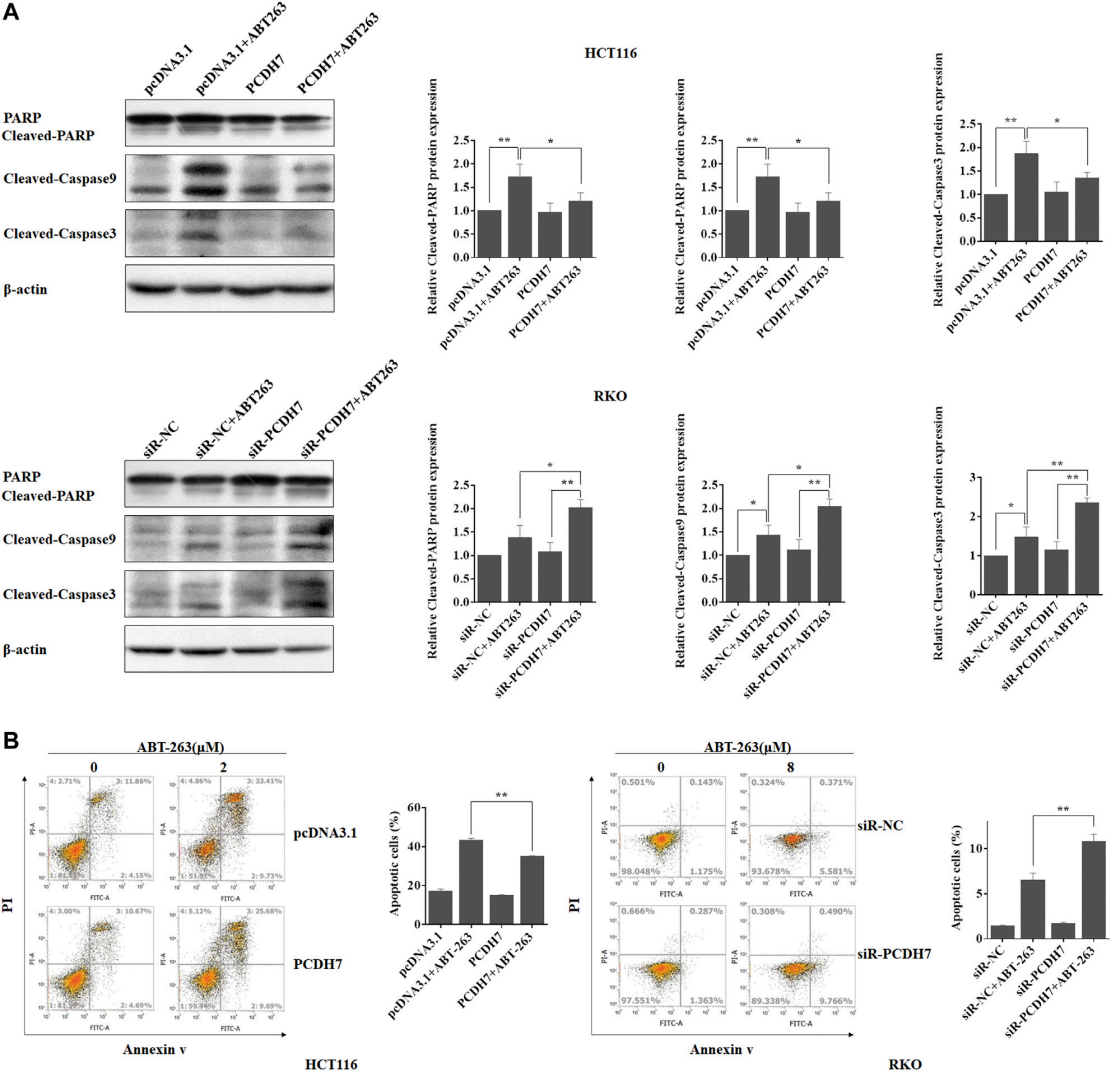
PCDH7 promoted drug resistance to CRC cells to ABT-263 by repressing apoptosis. **(A)** The cleavage of caspase 3, caspase 9, and PAPR was detected by western blot experiment in HCT116 and RKO cells treated with ABT-263 under PCDH7 overexpression or not, respectively. Representative data was shown. **(B)** HCT116 and RKO cells were treated with ABT-263 under PCDH7 overexpression or silencing, respectively. Apoptosis was examined by using flow cytometry with Annexin V-FITC/PI staining. Data are presented as mean ± SD for three separate experiments. *p < 0.05; **p < 0.01. vs. control.

Given that upregulation of PCDH7 increased CRC cell survival in response to ABT-263, an effective cell apoptosis inducer, we next determined whether the function of PCDH7 resulted from its anti-apoptotic effect. We further detected the regulation of PCDH7 on ABT-263-induced cleavage of caspase 3, caspase 9, and PARP (18). As shown in Figure 6A, ABT-263 treatment effectively induced the cleavage of caspase 3, caspase 9, and PAPR in both HCT116 and RKO cells. Surprisingly, cleaved-caspase 3, cleaved-caspase 9, and cleaved PAPR levels were significantly reduced following PCDH7 overexpression in HCT116 cells treated with ABT-263 (Figure 6A, upper panel). More interestingly, the levels of cleaved-caspase 3, cleaved-caspase 9, and cleaved PAPR obviously increased with the silencing of PCDH7 in RKO cells treated with ABT-263 (Figure 6A, down panel). These results prompted us to quantify the apoptosis of CRC cells treated with ABT-263 under overexpression or silencing of PCDH7 using Annexin V-FITC/PI staining. The percentage of ABT-263-induced apoptotic HCT116 cells increased to approximately 40% from nearly 17% after ABT263 treatment, while it was reduced to approximately 30% in PCDH7-overexpressed cells (Figure 6B, left panel). In RKO cells, the percentage of apoptotic cells increased from nearly 6% to 10% as a result of PCDH7 silencing in RKO cells under the ABT-263 treatment (Figure 6B, right panel). Hence, we proposed that higher PCDH7 levels would help colorectal cells suppress apoptosis and facilitate the survival of these cells during chemotherapeutic treatment.

### 3.6 The potential targets of the PCDH7 underlying molecular mechanism of its anti-apoptosis function

**FIGURE 7:**
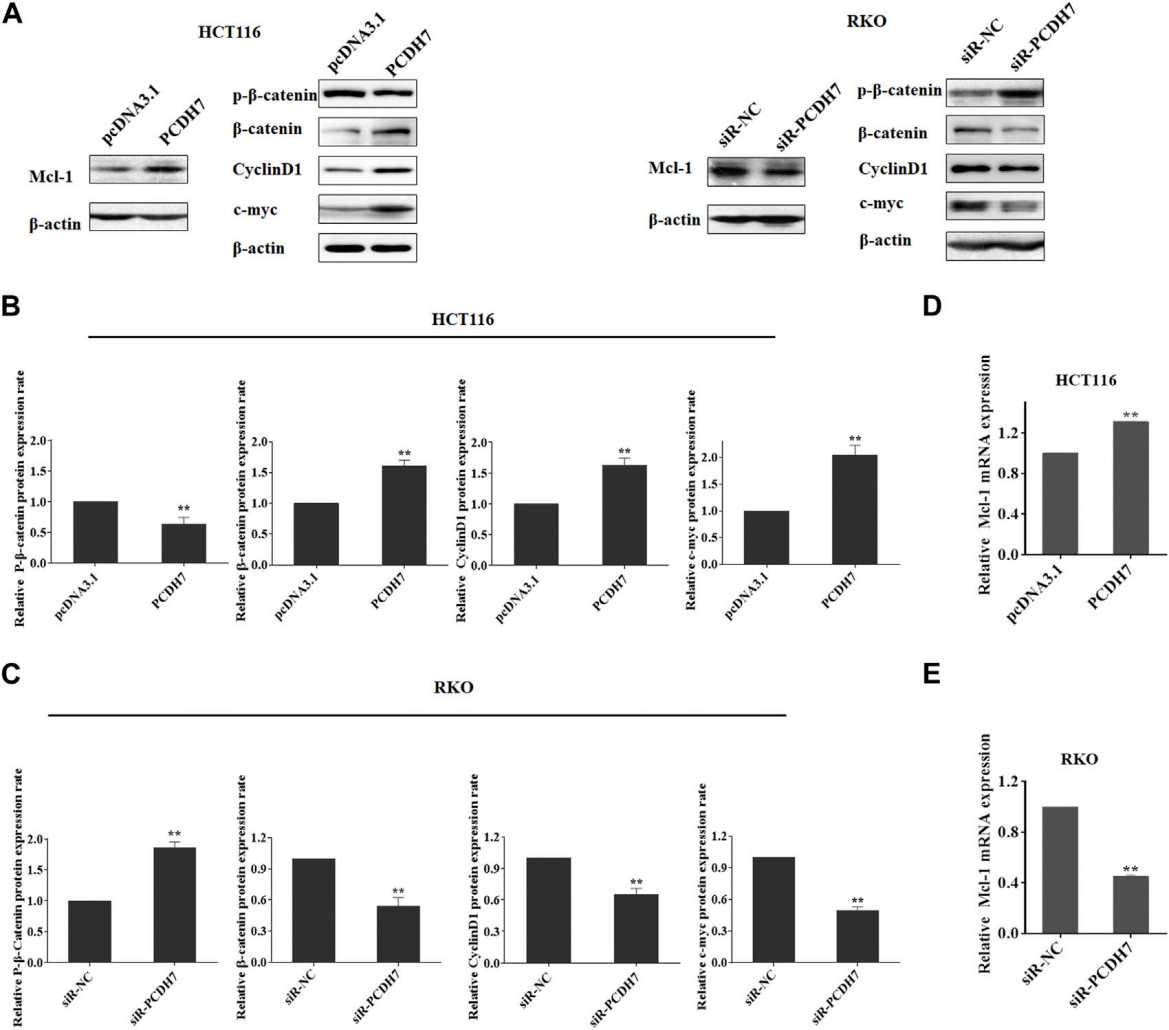
Role of PCDH7 in Mcl-1 expression and Wnt/β-catenin signaling pathway activation. **(A–C)** Western blot assays were performed to detect the protein expression of Mcl-1, p-β-catenin, β-catenin, cyclin D1, and c-Myc in HCT116 cells with PCDH7 overexpression and in RKO cells with PCDH7 knockdown, respectively. **(D, E)** Real-time qPCR experiments were performed to detect the mRNA expression of Mcl-1 in HCT116 cells with PCDH7 overexpression and in RKO cells with PCDH7 knockdown, respectively. Data are presented as mean ± SD for three separate experiments. **p < 0.01 *vs.* control.

Subsequently, we attempted to identify the potential targets of PCDH7 in colorectal cancer cells to further elucidate the underlying mechanism of its role in the development of drug resistance in CRC. Considering that ABT-263 is an inhibitor specific to anti-apoptotic BCL-2 family members, especially Mcl‐1 (Timucin et al., 2019), we first determined whether Mcl-1 could be affected by PCDH7 using Western blotting. As anticipated, the protein expression of Mcl-1 was upregulated following PCDH7 overexpression in HCT116 cells and downregulated after PCDH7 knockdown in RKO cells, which may partially explain the anti-apoptotic effect exerted by PCDH7 on colorectal cancer cells (Figures 7A–C). Additionally, we further evaluated the Wnt/β-catenin signaling pathway considering the massive evidence showing a correlation with chemoresistance in colorectal cancer (Su et al., 2015; Bahrami et al., 2017). Interestingly, the downregulation of P-β-catenin and the upregulation of β-catenin and its downstream target cyclin D1 and c-Myc were observed in PCDH7-overexpressed HCT116 cells, while all of these were completely reversed in PCDH7-silenced RKO cells (Figures 7A–C). Finally, real-time PCR assays indicated that the levels of Mcl-1 mRNA were upregulated in HCT116 cells with PCDH7 overexpression, while it was inhibited in RKO cells with PCDH7 silencing (Figures 7D, E).

### 3.7 PCDH7 mediated the drug resistance of CRC *via* upregulating Mcl-1

**FIGURE 8:**
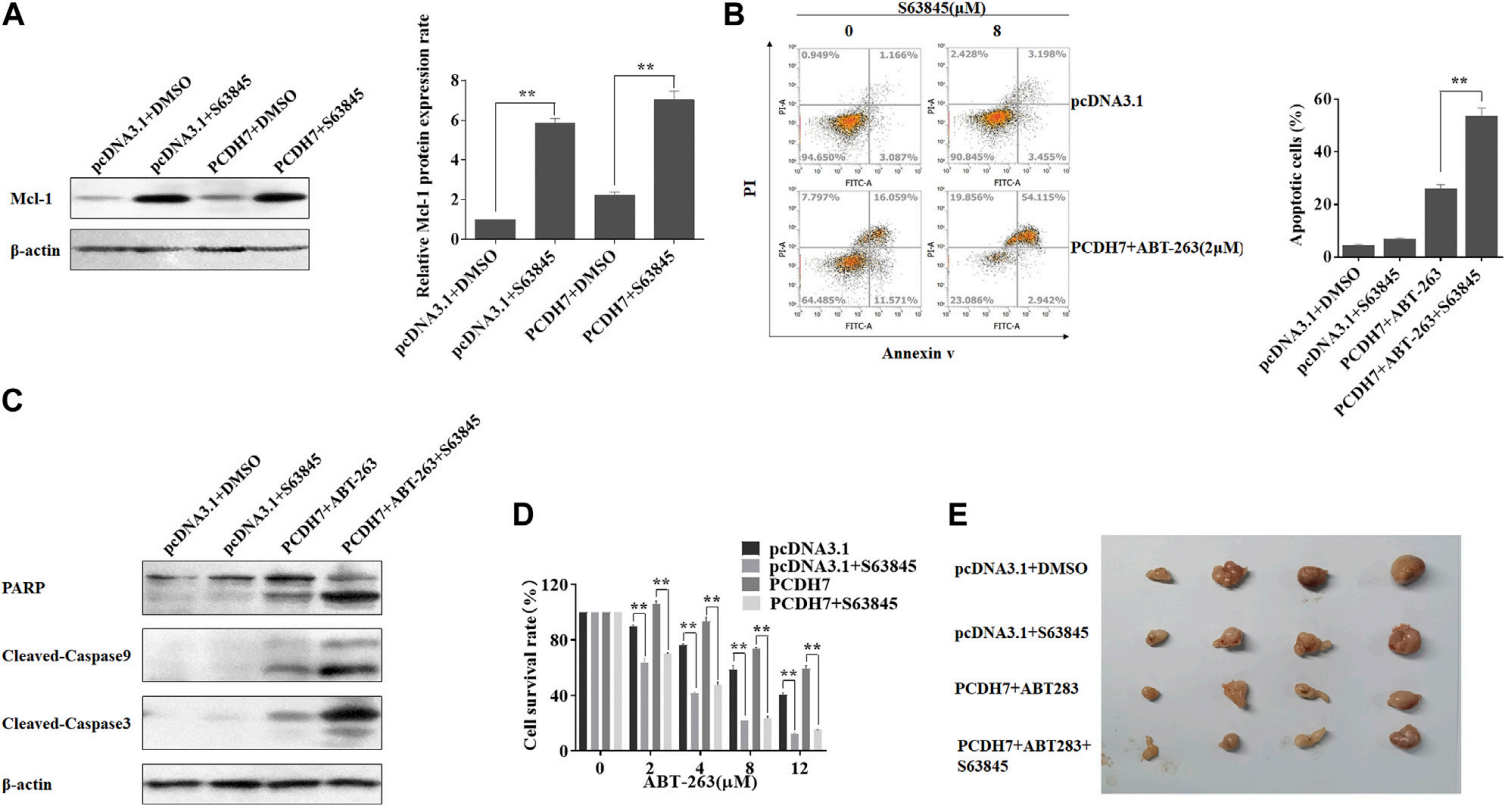
PCDH7 mediated the drug resistance of CRC *via* upregulating Mcl-1. **(A)** HCT116 cells were transfected with the PCDH7-overexpressed plasmid or treated with 8 μM S63845 for 24 h, and Mcl-1 protein expression was examined by Western blot assay. **(B)** HCT116 cells were transfected with the PCDH7 overexpressed plasmid combined with 2 μM ABT-263 with or without the treatment of 8 μM S63845 for 24 h. Cell apoptosis was determined using flow cytometry with Annexin V-FITC/PI staining. **(C)** Cleavage of PARP, caspase-3, and caspase-9 in HCT116 cells treated similarly in **(B)** was examined by Western blot assay. **(D)** HCT116 cells were transfected with the PCDH7 overexpressed plasmid with or without S63845 treatment, followed by the incubation of the indicated concentrations of ABT-263; MTT assay was performed to determine the cell viability. **(E)** HCT116 cells stably expressed PCDH7, or the control cells were used to generate xenografts in nude mice. The mice were treated as indicated, and the tumors were presented. Data are presented as mean ± SD for three separate experiments. **p < 0.01 *vs.* control.

To further confirm that PCDH7 affected colorectal cancer chemoresistance mainly through Mcl-1 expression, we applied an Mcl-1 inhibitor, S63845, in HCT116 cells coupled with PCDH7 overexpression. Surprisingly, Mcl-1 expression was dramatically upregulated in response to S63845 treatment compared to that in control, which may be a normal response to stress (Figure 8A). S63845 effectively attenuated the inhibitory effect of PCDH7 on ABT-263-induced apoptosis in HCT116 cells (Figure 8B, from approximately 27%–57%). This function was further supported by the increased cleavage of PARP, caspase-3, and caspase-9 in HCT116 cells treated with S63845 in combination with PCDH7 overexpression and ABT-263 treatment (Figure 8C). Moreover, S63845 treatment alone not only suppressed the proliferation of HCT116 cells but also effectively reversed the protective role of PCDH7 on the cytotoxicity of ABT-263 (Figure 8D). Finally, we tested our hypothesis in a xenograft model in nude mice. As shown in Figure 8E, although S63845 alone only slightly inhibited tumor growth, it effectively enhanced the anti-cancerous role of ABT-263 under PCDH7 overexpression. Collectively, PCDH7 might mediate drug resistance in CRC by upregulating Mcl-1, at least partially.

## 4 Discussion

In the present study, we characterized the abundance of PCDH7 in CRC cells correlated with their metastasis and chemoresistance. Here, we observed that upregulation of PCDH7 in colorectal cancer cells strongly promoted the activation of the Wnt/β-catenin signaling pathway, including the upregulation of catenin protein expression, which finally resulted in the expression of c-Myc, a widely reported protein essential for cell proliferation. These results partially explain the effects of PCDH7 on colorectal cancer cells.

There have been many studies showing that PCDH7 is abnormally expressed in various cancers and has a carcinogenic or anti-tumor effect (Terry et al., 2006; Cao et al., 2017; Liu et al., 2019). However, few studies have focused on the expression and role of individual PCDH7s in cancer. Previous studies have found that PCDH7 expression was significantly upregulated in human non-small cell lung cancer (NSCLC) (Zhou et al., 2017). PCDH7 was significantly downregulated in non-muscle invasive bladder cancer (NMIBC), and Cox analysis revealed that PCDH7 can be used as an independent predictor of NMIBC (13). The aforementioned investigations demonstrate that PCDH7 plays a role in various cancers. Arising from the bottleneck we encountered in developing effective inhibitors specific for RAS family proteins and the high proportion of resistance in response to *BRAF* inhibitors in colorectal clinic chemotherapy, there is an urgent need to explore the intrinsic molecular mechanism to provide a strategy for the combination of chemotherapeutics or identify a novel target for developing new inhibitors. In addition to modulating cell proliferation, we showed that PCDH7 would facilitate colorectal cancer cell survival under treatment with ABT-263 or cisplatin, which could induce apoptosis in various cancer cells by targeting anti-apoptotic proteins, which provides a scope for its development as a potential chemotherapeutic drug. Thus, PCDH7 was presumed to play an important role in chemo-treatment-induced apoptosis and was further revealed to positively modulate Mcl-1 expression, which is an anti-apoptotic protein belonging to the BCL-2 family, that results in higher cell viability in colorectal cancer with enhanced PCDH7 expression. Additionally, the combination of the Mcl-1 inhibitor S63845 with ABT-263 can eliminate chemoresistance caused by abundant PCDH7 in colorectal cancer cells. Our study provides strong evidence of the benefits of combining S63845 and ABT-263 for CRC, which is resistant to ABT-263 alone or with high levels of PCDH7.

Although numerous previous reports have illustrated the promotion of lung and breast cancer cell migration and invasion by PCDH7 (Chen et al., 2021), the increase in this protein inhibits cell migration and invasion in gastric cancer cells. However, a recent study has shown that P7 could promote lung metastasis of colorectal cancer (Liu et al., 2022), which seems contrary to our results, but we consider that this is related to different cell lines and experimental systems. This study showed that MCL-1 expression was upregulated after P7 overexpression, and the related molecules of the Wnt/β-catenin pathway were also upregulated. We speculated that P7 regulated MCL-1 through the Wnt/β-catenin pathway, thereby inhibiting apoptosis and promoting drug resistance of tumor cells. In these previous studies, the downregulation of E-cadherin expression followed by PCDH7 depletion was believed to contribute to an increased potential in the migration and invasion of gastric cancer cells (Chen et al., 2017). The contradictory effects of PCDH7 in different cancer types suggest that PCDH7 might play different roles through different molecular mechanisms. Although PCDH7 is a member of the protocadherin family, which is a subfamily of cadherins known to regulate epithelial-to-mesenchymal transition (EMT) of tumor metastasis during cancer progression, the related study of the detailed mechanism by which PCDH7 affects colorectal cancer metastasis is too little and needs further investigation in CRC. In conclusion, colorectal cells with high PCDH7 expression showed stronger chemoresistance to apoptosis induction. Because the Mcl-1 and Wnt/β-catenin signaling pathways were molecularly identified as regulatory targets of PCDH7, we discovered a combination of the Mcl-1 inhibitor S63845 and ABT-263 as a novel chemotherapy treatment option for PCDH7-high colorectal cancer.

## Data Availability

The original contributions presented in the study are included in the article/Supplementary Material; further inquiries can be directed to the corresponding authors.

## Abbreviations

ATCC, American Type Culture Collection; DMEM, Dulbecco’s modified Eagle’s medium; EMT, epithelial-to-mesenchymal transition; FBS, fetal bovine serum; protocadherin-7, PCDH7.
